# Mistranslation-associated perturbations of proteostasis do not promote accumulation of amyloid beta and plaque deposition in aged mouse brain

**DOI:** 10.1007/s00018-023-05031-z

**Published:** 2023-11-27

**Authors:** Harshitha Santhosh Kumar, James Moore, Adrian C. Steiner, Emmanuel Sotirakis, Benjamin Schärli, Patricia Isnard-Petit, Kader Thiam, David P. Wolfer, Erik C. Böttger

**Affiliations:** 1 Institute of Medical Microbiology, University of Zurich, Zurich, Switzerland; 2 Institute of Human Movement Sciences and Sport, D-HEST, ETH Zurich, Zurich, Switzerland; 3 genOway, Lyon, France; 4 Institute of Anatomy, University of Zurich, Zurich, Switzerland

**Keywords:** Proteostasis, Neurodegenerative disease, Alzheimer’s disease, Error-prone translation, Protein misfolding, Amyloid beta

## Abstract

A common perception in age-related neurodegenerative diseases posits that a decline in proteostasis is key to the accumulation of neuropathogenic proteins, such as amyloid beta (Aβ), and the development of sporadic Alzheimer’s disease (AD). To experimentally challenge the role of protein homeostasis in the accumulation of Alzheimer’s associated protein Aβ and levels of associated Tau phosphorylation, we disturbed proteostasis in single APP knock-in mouse models of AD building upon Rps9 D95N, a recently identified mammalian *ram* mutation which confers heightened levels of error-prone translation together with an increased propensity for random protein aggregation and which is associated with accelerated aging. We crossed the Rps9 D95N mutation into knock-in mice expressing humanized Aβ with different combinations of pathogenic mutations (wild-type, NL, NL-F, NL-G-F) causing a stepwise and quantifiable allele-dependent increase in the development of Aβ accumulation, levels of phosphorylated Tau, and neuropathology. Surprisingly, the misfolding-prone environment of the Rps9 D95N *ram* mutation did not affect Aβ accumulation and plaque formation, nor the level of phosphorylated Tau in any of the humanized APP knock-in lines. Our findings indicate that a misfolding-prone environment induced by error-prone translation with its inherent perturbations in protein homeostasis has little impact on the accumulation of pathogenic Aβ, plaque formation and associated phosphorylated Tau.

**Supplementary Information:**

The online version contains supplementary material available at 10.1007/s00018-023-05031-z.

## Introduction

The number of people worldwide suffering from neurodegenerative diseases exceeds that of people with cancer and is likely to increase even more over the next decades [18]. Many different disorders can cause neurodegeneration, but by far the most prevalent is Alzheimer’s disease (AD), a progressive and debilitating condition with no available cure to date [10]. In rare cases, AD is familial and dominantly inherited, with the misprocessing of amyloid precursor protein (APP) causing extensive deposition of extracellular amyloid beta (Aβ) plaques in the brain and early disease onset [2, 8]. However, late-onset (> 65 years) sporadic AD accounts for 95% of total cases [59], with age as the primary risk factor. After decades of the prevailing amyloid cascade hypothesis [21], the sole causative role of Aβ is coming under scrutiny [14, 16, 42]. Growing evidence of the pathological heterogeneity of sporadic AD and poor explanations for different comorbidities [1] suggests a more varied and complex pathogenesis with multiple contributing factors in addition to Aβ. The mixed outcomes of drug trials targeting Aβ testify to critical gaps in understanding AD pathogenesis [40, 56, 57].

Parsing out the role of different age-related changes in the development of AD is a challenge. Importantly, protein homeostasis (proteostasis) has been observed to decrease with age [25] and the accumulation of misfolded and aggregated proteins is a hallmark of aging [12, 35, 36]. The apparent age-related decline in proteostasis has led to the common perception that alterations in the buffering capacity of the proteostasis network contribute critically to the pathogenesis of age-related AD by allowing Aβ to accumulate [19, 25, 41]. However, the precise role of the proteostasis network in preventing AD is far from clear. In addition, there is mixed evidence for a causative role of proteostasis networks in the development of AD from genetic screens in model organisms [9], family-based genetic linkage analysis [3, 15, 24], and genome-wide association studies (GWAS) [7].

Given our incomplete understanding of the fundamental proteostatic mechanisms involved in Aβ deposition, we wished to address the role of perturbations in proteostasis in the accumulation of Aβ in knock-in mouse models expressing humanized pathogenic APP alleles. We reasoned that the recent availability of ribosomal ambiguity (*ram*) knock-in mice with heightened levels of error-prone translation and accelerated aging [48] should provide a suitable model to experimentally assess whether a misfolding-prone environment with its inherently increased propensity for protein aggregation and proteostasis decline affects the accumulation and aggregation of Aβ and the formation of Aβ plaques. As is characteristic for *ram* mutations, Rps9D95N confers misreading of near-cognate, but not non-cognate codons, resulting in elevated levels of mistranslation [48]. Towards this end, we introduced the Rps9 D95N *ram* mutation into APP knock-in mice which express humanized Aβ, both wild-type Aβ and various combinations of pathogenic Aβ mutations [45]. Surprisingly, we found that the presence of the Rps9 D95N mutation, while accelerating several age-related pathologies, did not affect the accumulation of Aβ or the formation of Aβ plaques in mouse brain expressing various pathogenic Aβ alleles, nor the formation of phosphorylated Tau species, suggesting that perturbations in proteostasis contribute little to the development of pathogenic Aβ aggregates.

## Materials and methods

### Generation of humanized APP knock-in mice

The mouse models were designed and developed by genOway, France, to enable expression of humanized Aβ (wild-type, NL, NL-F or NL-G-F) while maintaining a typical mouse APP expression pattern. The humanized APP (humAPP) mouse models were generated by knock-in using targeting vectors displaying the human Aβ sequence (either wild-type or harboring the NL, NL-F or NL-G-F mutations) and a neomycin selection cassette flanked by *loxP* sites. The homology arms were isolated by cloning from C57BL/6N mouse genomic DNA which is isogenic to the ES cell line used for homologous recombination. Mouse Aβ humanization was performed by humanizing the amino acids that are different between mouse and human sequences: G676R, F681Y and R684H within mouse exon 16 [45]. We also humanized the complete nucleic acid sequence of APP exons 16 and 17 to reflect the rare codons found in human APP exons 16 and 17, where the APP Swedish, Iberian and Arctic mutations are located (Supplementary Fig. 1). Rare codons are more prone to mistranslation and co-translational folding problems [30, 33] and introducing the human rare codons into mouse APP is expected to better reflect the relevant physiological rates of translation accuracy and folding of human APP. Four models were developed based on the humAPP sequence: wild type, NL, NL-F and NL-G-F. The integrity of the targeting vector was assessed by full sequencing.

As APP is known to be affected by alternative splicing [52], the impact of the combined mutations on splicing was measured in vitro in NIH-3T3 cells using qRT-PCR and validated by sequencing. Vectors were generated with and without humanized intron regions surrounding exons 16 and 17 to investigate potential splicing effects of exon humanization. Correctly spliced APP was produced in all genetic combinations tested (Supplementary Fig. 2a). However, the presence of humanized introns increased the production of mRNA from APP with humanized exons, as compared to humanized exons with mouse introns. Sequencing also revealed aberrant splice variants produced from constructs containing humanized exons and mouse introns, but none from constructs with humanized introns. Consequently, targeting vectors with humanized introns flanking humanized exons were used to generate mutant mice.

Linearized targeting vector was transfected into C57BL/6N ES cells according to genOway’s standard electroporation procedures. G-418 resistant ES cell clones were isolated, amplified, duplicated, and genotyped by PCR (Supplementary Fig. 2b), and the whole recombined locus was sequenced to confirm the absence of genetic alteration. Recombined ES cell clones were microinjected into mouse blastocysts, which gave rise to male chimeras with a significant recombined ES cell contribution. Breeding was established with a C57BL/6N Cre deleter line and produced the humAPP knock-in models devoid of the neomycin cassette (Supplementary Fig. 2c). This resulted in the following four heterozygous mouse lines expressing humanized Aβ: APP^WT^ (wild-type), APP^NL^ (Swedish mutation), APP^NL−F^ (Swedish mutation combined with Iberian mutation), and APP^NL−G−F^ (combination of Swedish, Iberian, and Arctic mutations). Heterozygous mice were genotyped by PCR using a trio of primers (Rev-common 5′-TACAAATCAGAAATGAACGTCTAGGTTCCG-3′, Fwd-1 5′-ACTGCATGTTTCCAAGTGGGAATTAAGAC-3′, Fwd-2 5′CCTGGGTGCCATGAATTTAATGTGC-3′; wild type amplicon: 197-bp, humanized amplicon: 398-bp). A subset of heterozygous animals was further subjected to full sequencing of the knock-in locus, including the homology arms, and a few kbs upstream and downstream of the homology arms.

Heterozygous APP mutants were then mated among themselves (APP^WT^, APP^NL^, APP^NL−F^, APP^NL−G−F^) and three of the lines (APP^WT^, APP^NL^, APP^NL−F^) were crossed with heterozygous Rps9 D95N mice [48] to obtain homozygous APP mutant mice and heterozygous APP and Rps9 D95N mice, respectively. The homozygous APP mutant mice and heterozygous APP and Rps9 D95N mice were then further crossed to produce double mutant lines with a homozygous APP mutation and heterozygous Rps9 D95N mutation. The presence of mutations was checked by PCR amplification and sequencing after each step. Finally, the following lines were available for analysis: humAPP^WT/WT^/Rps9 WT; humAPP^WT/WT^/Rps9 D95N; humAPP^NL/NL^/Rps9 WT; humAPP^NL/NL^/Rps9 D95N; humAPP^NL−F/NL−F^/Rps9 WT; humAPP^NL−F/NL−F^/Rps9 D95N; humAPP^NL−G−F/NL−G−F^/Rps9 WT.

### Harvesting

Approximately balanced numbers of male and female animals were euthanized at the age of 15 months by CO_2_ inhalation and brains were harvested immediately. Left hemispheres were divided into telencephalon/diencephalon and brainstem/cerebellum, snap-frozen in liquid nitrogen and subsequently used for biochemical analyses, while the right hemispheres were immersion fixed in 4% PFA with picric acid and kept in the fixative at 4 °C for histopathology.

### Amyloid beta quantification using ELISA

The samples were prepared for ELISA as described previously [28]. In brief, deep frozen cortex was grounded and further homogenized on ice in 400 µL TBS buffer (20 mg frozen cortex, 50 mM Tris–HCl—pH7.6, 150 mM NaCl with Roche cOmplete Protease Inhibitor Cocktail (Roche)) using a motorized pestle. Protein concentrations were measured and normalized between samples using the Micro BCA Protein Assay Kit (Thermo Scientific). Samples were then centrifuged at 200,000 g for 20 min at 4 °C. The supernatant was used as the soluble fraction. Guanidine-HCl was added to the soluble fraction at 0.5 M final concentration before application to ELISA. The pellet was suspended in 6 M Guanidine-HCl buffer with Roche cOmplete Protease Inhibitor Cocktail (Roche) and sonicated for 3 min, followed by centrifugation at 200,000 g for 20 min at 4 °C to remove any debris. The supernatant was diluted 12 times in TBS buffer to reduce the concentration of Guanidine-HCl and used as the insoluble fraction. Commercial mouse Aβ ELISA kits were used to quantify Aβ40 (Thermo Scientific KHB3481) and Aβ42 (Thermo Scientific KHB3441) in the soluble and insoluble fractions, according to the manufacturer’s instructions. Samples with high concentration of the target protein were diluted to avoid detection saturation. Read values from each plate were normalized such that the mean value of humAPP^WT/WT^/Rps9 WT at each read was equal to 15 pg/ml for Aβ40 and 25 pg/ml for Aβ42. Statistical analysis was performed with all animals using GraphPad Prism 5.0 software; an unpaired Student’s t-test was used to estimate significance.

### Western blot

To detect APP, the lysate prepared with TBS buffer (50 mM Tris–HCl – pH 7.6, 150 mM NaCl with Roche cOmplete Protease Inhibitor Cocktail (Roche)) for ELISA was boiled in SDS sample buffer for 5 min, resolved by SDS-PAGE and transferred to PVDF. For other proteins, deep frozen cortex from 15-month-old male and female animals was grounded and homogenized in urea lysis buffer (100 nM Tris, 10 mM magnesium acetate, 6 M urea, 2% SDS, 10 µM DTT) with Roche cOmplete Protease Inhibitor Cocktail (Roche) and HALTTM Phosphatase Inhibitor Cocktail (Thermo Scientific), and incubated for 3 h at room temperature. The resulting homogenate was centrifuged at 13,000 g for 10 min at 4 °C to remove cell debris. The supernatant was collected and protein concentration was measured using the Micro BCA Protein Assay Kit (Thermo Scientific). Samples were boiled in SDS sample buffer for 5 min, resolved by SDS-PAGE and transferred to PVDF membranes (Bio-Rad). The primary antibodies used were: HRP anti-GAPDH (Abcam ab9482), eIF2α (Cell Signaling Technology 9722S), Phospho-eIF2α (Cell Signaling Technology 3398S), ATF-4 (Cell Signaling Technology 11815S), GADD34 (Thermo Scientific PA1-139), BACE D10E5 (Cell Signaling Technology 5606S), APP (Sigma-Aldrich A8717), Tau (Abcam ab76128), Phospho Tau S396 (Abcam ab109390), and Phospho Tau AT8 (Thermo Scientific MN1020) Secondary antibodies used were: goat anti-rabbit HRP (Abcam ab6721) and rabbit anti-mouse HRP (Abcam ab6728). Statistical analysis was performed with male and female densitometry values using GraphPad Prism 5.0 software. To combine densitometry values from two different gels, values were normalized such that the mean of the relative wild-type control was equal to one. An unpaired Student`s t test was used to estimate significance.

### Histopathology

The right brain hemispheres were transferred into a solution of 30% sucrose and sodium azide for 12–24 h for cryoprotection. They were cut in coronal orientation on a freezing microtome (Zeiss Hyrax S30, Thermo Fisher HM 430) to a section thickness of 40 or 50 μm. We collected 10 series of sections, placing 9 into Eppendorf tubes with cryoprotection solution for later processing and immediately mounting one in anatomical order as a reference series with a modified Giemsa stain [27] (Merck 109,204). Immunohistochemistry was performed on free-floating sections, with only sections containing parts of the hippocampus being selected. In order to maximize the number of independent observations in biochemical and neuropathological quantifications, we did not perfuse separate animals for histopathology. While reducing the total number of animals, using hemispheres of unperfused brains for immunohistochemistry unfortunately increases background and led to variable non-specific staining of blood vessels in some of the procedures.

For immunostaining of Aβ plaques, rinsing at the start and in between steps was performed in phosphate-buffered saline (PBS, 0.01 M, pH = 7.4). Endogenous peroxidase activity was inhibited with 0.6% H2O2 and 1% Triton X-100 (Sigma-Aldrich T8787) in PBS for 15 min. After pre-incubation in blocking buffer (2% normal horse serum, 2% BSA and 1% Triton X-100 in PBS) for 90 min at room temperature, sections were incubated overnight at 4 °C in primary antibody solution (Mouse anti-human Aβ (N) (82E1) 1:100, IBL 10323 1:100). On the next day, sections were transferred to secondary antibody solution (biotinylated horse anti-mouse 1:300, Vector BA-2000) for 30 min. Then, incubation in avidin–biotin complex solution (Vector PK-6100) was performed for 60 min (0.5% A, 0.5% B in Tris–HCl). Subsequently, 3,3-Diaminobenzidine (Sigma-Aldrich D4418) was applied as chromogen, sections were mounted on gelatin-coated glass slides, lightly counterstained with Giemsa’s solution (Merck 109,204) and dehydrated in alcohol solutions of increasing concentration (70%, 94%, and 2 × 100%, respectively). Finally, cover slips were added using Eukitt® mounting medium (Sigma-Aldrich 03989).

In order to assess microgliosis and astrocytosis as markers of neuroinflammation, we performed immunohistochemical staining with monoclonal antibodies against Iba1 and GFAP. Tris-Triton (Phosphate-buffered saline + 0.05% Triton X-100) was used for rinsing here. Epitope retrieval was performed in citrate buffer (pH = 6.0, Sigma-Aldrich C9999) diluted 1:10 in distilled water at 95° C for 40 min for Iba1 staining. For GFAP staining, we resorted to microwaving for 1 min at 900 watts instead, using the same solution. After cooling, 0.6% H_2_O_2_ in Tris-Triton was used for endogenous peroxidase inhibition for 15 min. Sections for GFAP staining were pre-incubated in a solution of Tris-Triton + 0.2% Triton X-100 + 2% normal horse serum + 0.1% BSA at room temperature for 1 h, whereas for Iba1 staining, we did not add BSA and chose normal goat serum to match the host species of the secondary antibody. Then, sections were transferred to primary antibody solution, which consisted of pre-incubation buffer and Rabbit anti-Iba1 1:3000 (Wako 019-19,741) and mouse anti-GFAP antibody 1:2000 (Merck Millipore IF03L), respectively, for overnight incubation at 4 °C. Sections were rinsed again, with the medium being switched to Tris-buffered-saline (TBS) from this point onward. Sections were incubated in secondary antibody solution for 40 min at room temperature: TBS + 0.1% BSA + 2% normal horse serum + 1:300 biotinylated horse anti-mouse (Vector BA-2000) for GFAP staining and TBS + 0.1% BSA + 2% normal goat serum + 1:300 biotinylated goat anti-rabbit (Vector BA-1000) for Iba1 staining were used. Further processing was performed in the same manner as described for Aβ staining, with the exception of ABC incubation: here, we used a concentration of 1% for each reagent and incubated for 20 min in TBS instead of Tris–HCl.

For double staining of Aβ and GFAP, we first stained sections for Aβ as described above. Then, we applied an avidin/biotin blocking kit (Vector SP2001) without dilution, treated sections with 3% H_2_O_2_ in TBS for 15 min and pre-incubated them with 0.25% Triton X-100, 5% normal horse serum and 4% BSA in TBS for 1 h at room temperature. Incubation with second primary antibody solution was performed with mouse anti-GFAP antibody 1:2000 (Merck Millipore IF03L) in pre-incubation buffer and sections were left overnight at 4 °C. After incubation in secondary antibody solution (1:300 biotinylated horse anti-mouse + 5% normal horse serum in TBS) for 30 min and incubation in ABC solution for 20 min, Vector SG peroxidase substrate kit (Vector SK-4700) was used as the second chromogen to obtain a blackish-blue stain for GFAP. Sections were then prepared as previously described, but Giemsa counterstaining was omitted due to its color spectrum overlapping with that of the Vector SG dye. We double-stained Aβ and Iba1 in the same fashion, and adjusted primary antibody concentrations to optimize contrast (Aβ 1:1000, Iba1 1:1000).

### Quantification of plaque load and glial reaction

Quantification was performed by investigators who were unaware of animal genotype and well trained to distinguish between labeled target structures and non-specific staining of blood vessels. We analyzed load and distribution of Amyloid β plaques using the software Stereo Investigator® (MBF Bioscience, version 2020.1.2). Hippocampal area estimates were obtained using a Cavalieri estimator probe [49] on 5–9 sections per animal in anatomical order, with one series—thus every tenth section cut—analyzed. A nucleator probe [20] at 200 × magnification was then applied to the same sections to obtain an estimate of plaque area. The percentage of hippocampal area covered in plaques served as a measure of plaque load. A repeated-measures ANOVA was performed on the results. The degree of astrocytosis as indicated by number, distribution and morphology of GFAP-positive astrocytes was investigated using a rank-based approach. Photomicrographs of hippocampal and cortical regions (one for each subject, 38 images per region) were taken at approximately the same location on the rostrocaudal axis for every animal. Then, 5 experienced investigators were asked to rank the images on a scale from 1 (weakest labeling) to 38 (strongest labeling) according to two different categories of GFAP-signal while ignoring non-specific staining of blood vessels: Number and intensity of localized clusters of activated astrocytes and level of general diffuse astrocyte activation. To examine microgliosis, the same approach was used and the ranking was again divided into an assessment of cluster-like signal and one of overall microglial activity. Means of observer rankings were then analyzed with a Kruskal–Wallis test. R software version 4.2.1 (http://www.r-project.org) [44] complemented by the ggplot2 package was used for all statistical analyses and visualization of the data.

### Ethical animal research statement

All experiments performed on *M. musculus* C57BL/6 complied with ethical regulations for animal testing and research and were approved by the Veterinary Office of the Canton of Zurich (licenses ZH207/2019, ZH060/2021).

## Results

### Effect of APP mutations on Aβ accumulation and plaque formation

**Fig. 1:**
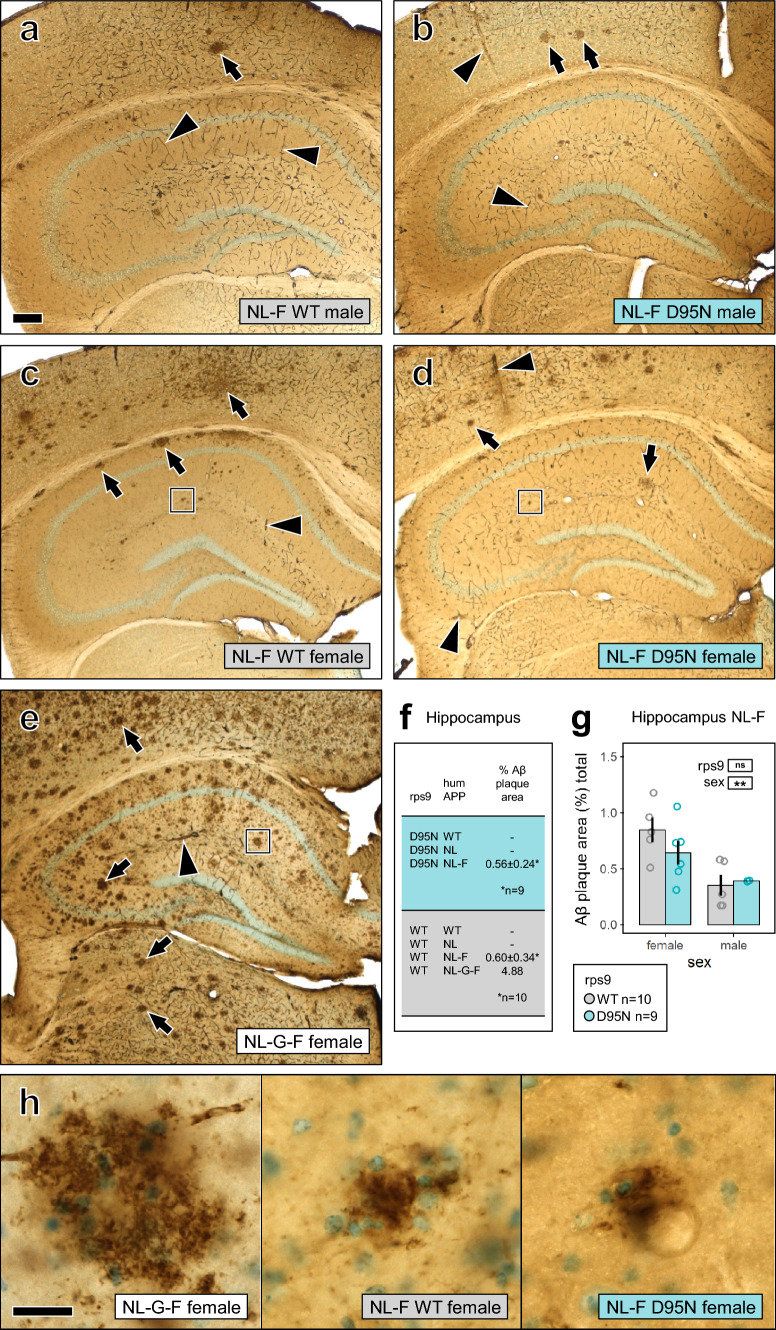
Rps9 D95N mutation does not affect Aβ plaque deposition. Immunohistochemical labelling of Aβ plaques is shown in the dorsal hippocampus of humAPP^NL−F/NL−F^ mice without (**a**, **c**) and with the Rps9 D95N mutation (**b**, **d**). A humAPP^NL−G−F/NL−G−F^ mouse is shown as reference in (**e**). Quantification of plaque area in humAPP^NL−F/NL−F^ mice by stereology is shown in (**f**–**g**). An overview of all genotypes is given in (**f**). There were no plaques in humAPP^WT/WT^ and humAPP^NL/NL^ mice, one humAPP^NL−G−F/NL−G−F^ mouse was quantified as reference. Statistical examination of plaque area in humAPP^NL−F/NL−F^ mice is depicted in (**g**) (mean and SE, ANOVA with between factors rps9 genotype and sex, number of brains: Rps9 WT 5 females / 5 males, D95N 6 females / 3 males). Female mice had more plaques than males but there was no evidence for an enhancing effect of the Rps9 D95N mutation on Aβ plaque deposition (genotype F1,15 = 0.050 ns, sex F1,15 = 14.02 *p* = .0020, genotype x sex F1,15 = 1.465 ns). Close-ups of the boxed areas in (**c**–**e**) are shown in (**h**). Aβ plaques appear in brown, Giemsa counterstain in light blue. Arrows point at plaques, arrowheads at non-specific staining of blood vessels due to the use of tissue from unperfused animals. Scale bars 200 μm (**a**–**e**), 20 μm (**h**)

We determined Aβ accumulation by measuring levels of soluble and insoluble Aβ40 and Aβ42 in mouse brain cortices using ELISA and further assessed the presence of Aβ plaques by immunohistochemistry in brain sections. Consistent with published reports on APP knock-in mice expressing humanized Aβ [34, 45], we observed an APP allele-dependent increase in Aβ accumulation (Supplementary Fig. 3a–d). In detail, compared to humanized APP wild-type, the NL mutation presented with slightly increased amounts of Aβ40, both soluble and insoluble, but NL had no effect on Aβ42 levels and as humanized APP wild-type showed complete absence of pathology (Fig. 1f, Supplementary Fig. 4a, c). In contrast, the NL-F combinational mutation showed considerably increased levels of soluble and insoluble Aβ42, as well as moderate plaque deposition in neocortex and hippocampus which was more pronounced in female mice (Fig. 1a, c, f, g). The combinational NL-G-F mutation came along with similar levels of soluble Aβ40 and Aβ42 as NL-F, but markedly increased levels of insoluble Aβ40 and Aβ42, and massive plaque deposition throughout the brain (Fig. 1e, f, h).

### Effect of APP mutations on phosphorylated Tau species

Aβ deposition has been shown to increase Tau phosphorylation [38, 58] and Tau hyperphosphorylation is a well-characterized pathological modification which contributes to Tau aggregation and the formation of neurofibrillary tangles (NFTs) in Alzheimer’s disease brains [43]. To assess pathological changes in Tau, we quantified the phosphorylation of Tau in the cortex by Western blot using the AT8 antibody (p-S202 and p-T205), classically used in post-mortem biochemical staging of Alzheimer’s disease brains to indicate abnormal Tau phosphorylation [5, 17]. We also measured Tau phosphorylation at S396, which has been shown to increase in late-stage Alzheimer’s disease [43]. We compared mice expressing humanized Aβ WT, Aβ NL-F and Aβ NL-G-F and found a genotype dependent increase in AT8 phosphorylation, increased in NL-F relative to WT and further increasing in NL-G-F mice (Supplementary Fig. 3e). Phosphorylation at S396 and total Tau levels were unaffected. Thus, heightened synthesis of pathological Aβ came along with a progressive increase in AT8 phosphorylation. This observation supports previous observations that AT8 phosphorylation is increased in NL-G-F mice relative to WT [46].

### Effect of Rps9 D95N on Aβ accumulation and plaque formation

The stepwise and quantifiable APP allele-dependent increase in Aβ accumulation and corresponding plaque formation observed in mice expressing humanized Aβ WT, NL, NL-F, and NL-G-F provides a sensitive means to study the effect of disturbed proteostasis on Aβ accumulation, Tau phosphorylation, and plaque formation. To assess Aβ accumulation in an environment of disturbed proteostasis, we compared the levels of soluble and insoluble Aβ40 and Aβ42 by ELISA in the cortices of humAPP^WT/WT^, mutant humAPP^NL/NL^, and mutant humAPP^NL−F/NL−F^ in the presence and absence of the translation error-prone Rps9 D95N mutation. Mice expressing humAPP^NL−G−F/NL−G−F^ were used as a positive control for Aβ accumulation and pathology.

**Fig. 2:**
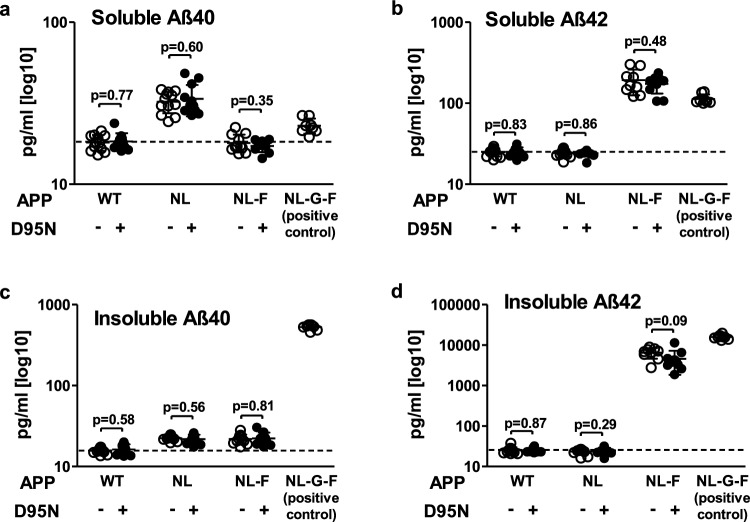
Rps9 D95N mutation has no detectable effect on levels of Aβ40, Aβ42 in the cortex of mice with different humAPP alleles. **a**–**d** The Rps9 D95N *ram* mutation was introduced into mice harboring different alleles of humAPP (humAPP^WT/WT^, humAPP^NL/NL^, humAPP^NL−F/NL−F^) to study the effect of Rps9 D95N in genetic backgrounds with higher propensity for Aβ production. humAPP^NL−G−F/NL−G−F^ mice were used as a positive control for insoluble Aβ production. **a** Soluble Aβ40. **b** Soluble Aβ42. **c** Insoluble Aβ40. **d** Insoluble Aβ42. (8 ≤ N ≤ 12) (Males 4 ≤ N ≤ 7; Females 4 ≤ N ≤ 6). Plots show mean, SD, and individual data points

To our surprise, we observed no differences in the levels of soluble or insoluble Aβ40 and Aβ42 between humAPP/Rps9 WT and humAPP/Rps9 D95N mice across the humAPP^WT/WT^, humAPP^NL/NL^, and humAPP^NL−F/NL−F^ genotypes (Fig. 2a–d). Close inspection of the data showed that there was very little variability in measurements of Aβ across individual mice within each genotype, and no obvious trends or tendencies in Aβ expression were observed between humAPP/Rps9 WT and humAPP/Rps9 D95N, supporting the robustness of the Aβ ELISA measurements with its absence of any minor difference induced by the Rps9 D95N mutation. To control for the potential confounding effects of variable APP expression, we measured total APP protein levels by western blot and observed no differences between humAPP/Rps9 WT and humAPP/Rps9 D95N (Supplementary Fig. 5). These observations indicate that the Rps9 D95N mutation has no impact on the accumulation of Aβ, even under conditions of considerably increased Aβ synthesis due to APP mutations. Deposition of Aβ plaques in presence of the Rps9 D95N mutation was investigated by immunohistochemistry. Mirroring the results of the independent assessment of Aβ accumulation by ELISA, the Rps9 D95N mutation did not induce any plaque deposition in brain sections of humAPP^WT/WT^ or mutant humAPP^NL/NL^ mice (Supplementary Fig. 4b, d), and humAPP^NL−F/NL−F^ mice showed similar moderate plaque deposition irrespective of presence or absence of the Rps9 D95N mutation (Fig. 1a–e). Quantification of plaque area fraction by stereology in the hippocampus confirmed our qualitative observations (Fig. 1f,g).

### Effect of Rps9 D95N on levels of phosphorylated Tau

**Fig. 3:**
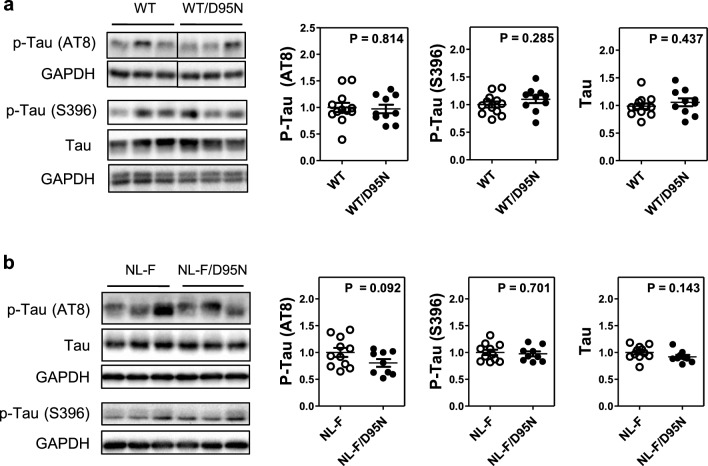
Rps9 D95N mutation has no detectable effect on levels of phosphorylated Tau in the cortex of mice with different humAPP alleles. **a** Representative western blot images and densitometry analysis of Tau and phosphorylated Tau proteins, comparing humAPP^WT/WT^/Rps9 WT with humAPP^WT/WT^/Rps9 D95N mice (10 ≤ N ≤ 12) (Males *N* = 6; Females 4 ≤ N ≤ 6). GAPDH was used as loading control. Densitometry depicts relative expression of target gene normalized to loading control. For comparison, the non D95N genotype was set as 1. *N* = number of mice in each comparison. Graph shows mean, SEM and individual data points. **b** Representative western blot images and densitometry analysis of Tau and phosphorylated Tau proteins comparing humAPP^NL−F/NL−F^/Rps9 WT with humAPP^NL−F/NL−F^/Rps9 D95N mice (9 ≤ N ≤ 11) (Males 4 ≤ N ≤ 6; Females N = 5). GAPDH was used as loading control. Densitometry depicts relative expression of target gene normalized to loading control. For comparison, the non-D95N genotype was set as 1. *N* = number of mice in each comparison. Graph shows mean, SEM and individual data points

We next investigated Tau phosphorylation in the same cortex tissue, comparing humAPP/Rps9 WT and humAPP/Rps9 D95N mice. Consistent with the Aβ measurements, there were no differences in the amounts of total or phosphorylated Tau AT8 or S396 in humAPP/Rps9 WT and humAPP/Rps9 D95N mice expressing humAPP^WT/WT^ (Fig. 3a) or humAPP^NL−F/NL−F^ (Fig. 3b). The absence of altered Tau phosphorylation induced by Rps9 D95N mice provides further evidence that the perturbations in proteostasis as present in Rps9 D95N mice do not amplify Aβ associated pathological changes.

### Effect of APP and Rps9 D95N mutations on neuroinflammation

**Fig. 4:**
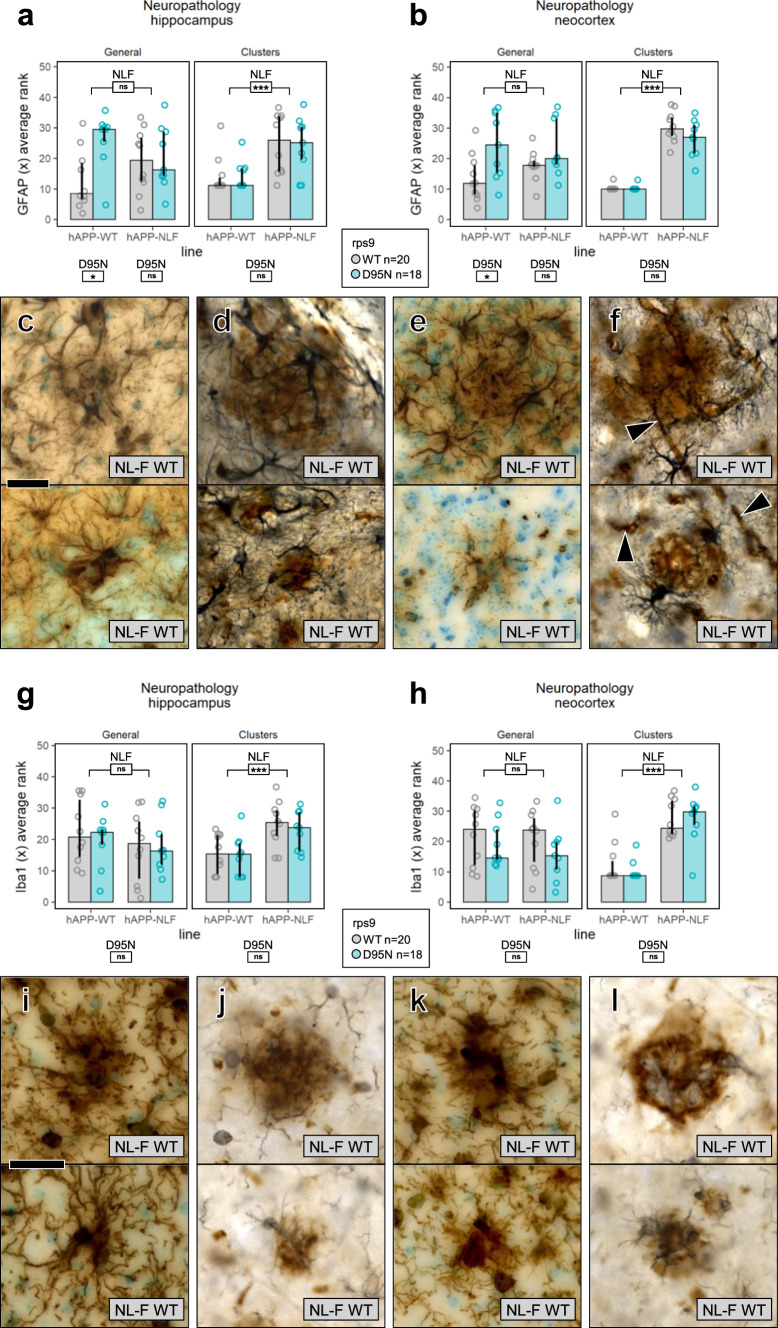
The Rps9 D95N mutation has no detectable effect on the formation of plaque-associated astro- and microglial clusters. **a–f** analysis of astroglial activation. **a** Rank scores of immunohistochemical GFAP labelling in dorsal hippocampus (median and interquartile range). 5 observers, blinded to genotypes, rank-sorted low magnification views of the dorsal hippocampus based on the intensity of general diffuse astrogliosis as well as on number and size of astroglial clusters. While clusters were only rarely seen in humAPP^WT/WT^ mice, they were clearly more numerous in humAPP^NL−F/NL−F^ mice (Kruskal–Wallis test on averaged ranks: *p* = .0006). The NLF mutations had no evident effect on general diffuse astrogliosis. The Rps9 D95N mutation was associated with general diffuse astrogliosis in humAPP^WT/WT^ mice but had no evident effect on astrocyte cluster formation. **b** Rank scores of GFAP labelling in neocortex. While clusters were virtually absent in humAPP^WT/WT^ mice, they were numerous in humAPP^NL−F/NL−F^ mice (Kruskal–Wallis test on averaged ranks: *p* < .0001). Examples of close-ups of large and small clusters of activated astrocytes in dorsal hippocampus (**c**) and neocortex (**e**) are shown with GFAP labeling in brown and Giemsa counterstain in light blue. The association of clusters with Aβ plaques in hippocampus (**d**) and neocortex (**f**) is visualized by double immunohistochemistry with GFAP labeled black and Aβ plaques stained brown. Arrowheads point at non-specific staining of blood vessels, all close-ups taken from humAPP^NL−F/NL−F^ Rps9 WT mice. **g**–**l** analysis of microglial activation. **g** Rank scores of immunohistochemical Iba1 labelling in dorsal hippocampus (median and interquartile range). 5 observers, blinded for genotypes, rank-sorted low magnification views of the dorsal hippocampus based on the intensity of general diffuse microgliosis as well as on number and size of microglial clusters. While few clusters were apparent in humAPP^WT/WT^ mice, they were clearly more numerous in humAPP^NL−F/NL−F^ mice (Kruskal–Wallis test on averaged ranks: *p* = .0004). The NLF mutations had no evident effect on general diffuse microgliosis. The Rps9 D95N mutation had no evident effect on microglial activation of either type. **h** Rank scores of Iba1 labelling in neocortex. While clusters were rarely detected in humAPP^WT/WT^ mice, they were numerous in humAPP^NL−F/NL−F^ mice (Kruskal–Wallis test on averaged ranks: *p* < .0001). Examples of close-ups of large and small clusters of activated microglia in dorsal hippocampus (**i**) and neocortex (**k**) are shown with Iba1 labeling in brown and Giemsa counterstain in light blue. The overlap of clusters with Aβ plaques in hippocampus (**j**) and neocortex (**l**) is visualized by double immunohistochemistry with Iba1 labeled black and Aβ plaques stained brown. All close-ups taken from humAPP^NL−F/NL−F^ Rps9 WT mice. Number of brains: humAPP^WT/WT^ Rps9 WT 5 female / 5 male, D95N 3 female / 6 male, humAPP^NL−F/NL−F^ Rps9 WT 5 female / 5 male, D95N 6 female / 3 male. Scale bars 25 μm (**c**–**f**) and (**i**–**l**)

Astroglial activation was assessed by GFAP immunohistochemistry in brain sections of humAPP^WT/WT^ and humAPP^NL−F/NL−F^ mice in presence and absence of the Rps9 D95N mutation. General diffuse astrogliosis (Fig. 4a-b; Supplementary Fig. 6a-d) and occurrence of clusters of activated astroglia (Fig. 4a–f) were quantified separately by ranking in 9–10 mice of each genotype combination in the dorsal hippocampus as well as in the parietal neocortex. While the amyloidogenic humAPP^NL−F/NL−F^ mutation strongly and significantly increased the occurrence of astroglial clusters in both hippocampus and neocortex (Fig. 4a–b), no effect of the Rps9 mutation on cluster formation was evident. Double immunohistochemistry for Aβ and GFAP in humAPP^NL−F/NL−F^ (Fig. 4d, f) mice confirmed that the majority of clusters of activated astrocytes were indeed associated with Aβ plaques [11, 39, 45]. No effect of the humAPP^NL−F/NL−F^ mutation on diffuse astrogliosis was evident, but in line with previous observations [6] diffuse astroglial activation was more pronounced in mice of the humAPP^WT/WT^ line carrying the Rps9 D95N mutation (Fig. 4a–b). In both examined regions, specific labeling of astrocytes could be distinguished reliably from non-specific staining of blood vessels (Fig. 4f, Supplementary Fig. 6d).

We next assessed microglial activation using Iba1 immunohistochemistry as marker for microgliosis (Fig. 4g–l; Supplementary Fig. 6e–h). Formation of clusters of activated microglia was clearly stronger in humAPP^NL−F/NL−F^ than in humAPP^WT/WT^ mice, in the hippocampus as well as in the neocortex, however, no effect of the Rps9 D95N mutation was evident (Fig. 4g–h). Double immunohistochemistry for Aβ and Iba1 in humAPP^NL−F/NL−F^ mice (Fig. 4j, l) confirmed that the majority of clusters of activated microglial cells overlapped with Aβ plaques [11, 45]. We did not observe an enhancing effect of either mutation on generalized diffuse activation of microglia (Fig. 4g–h).

### Effect of APP and Rps9 D95N mutations on integrated stress response

As a further characterization of the robustness of our model, we studied the integrated stress response (ISR). As in previous studies [23], we found that activation of ISR was not induced in humAPP^NL−G−F/NL−G−F^ mice relative to humAPP^WT/WT^, as determined by eIF2a phosphorylation and the expression of eIF2a targets GADD34 and ATF4 (Supplementary Fig. 7). Expression of the beta-site amyloid precursor protein-cleaving enzyme 1 (BACE1), a protease essential for A beta production in AD, was increased in APP^NL−G−F/NL−G−F^ mice, as reported previously [32]. In line with our previous finding of absent ISR activation in Rps9 D95N mutants [6], the presence of the Rps9 D95N *ram* mutation did not measurably augment ISR or BACE1 expression in humAPP^WT/WT^ mice (Supplementary Fig. 7b), nor in mice with the pathogenic humAPP NL-F allele (Supplementary Fig. 7c).

## Discussion

A long-standing hypothesis posits that a decline in proteostasis contributes to the development of age-associated NDDs and the formation of pathogenic Aβ plaques [19, 25, 41]. We here addressed this question experimentally by studying whether a misfolding-prone environment with disturbed proteostasis as mediated by heightened levels of error-prone translation affects APP processing and the accumulation of pathogenic Aβ. Towards this end, we crossed two established genetically modified mouse models, namely humanized APP knock-in mice carrying combinations of various amyloidogenic mutations (WT, NL, NL-F) [45] with mice carrying the translation error-prone Rps9 D95N *ram* mutation [6, 48]. We reasoned that the stepwise and quantifiable increase in Aβ accumulation and pathogenic plaque formation, associated with the different amyloidogenic mutations, should provide a sensitive means to test the hypothesis of impaired protein homeostasis fostering the accumulation of Aβ. However, while replicating established phenotypes of the original lines [6, 23, 45], our data obtained in double mutant lines show that the perturbations of protein homeostasis, as present in D95N mice, neither foster the accumulation of Aβ nor induce or promote Aβ plaque deposition and associated glial activation. While we can formally not rule out that the Rps9 D95N mutation may accelerate the development of AD-associated phenotypes in the humanized APP mice, we consider this possibility unlikely, as our analysis of 15 months old mice testifies to the complete absence of a Rps9 D95N mutation effect on AD-associated pathology. We conclude from this data that error-prone translation and concomitant proteostasis decline, while resulting in accelerated aging and the premature development of several age-related phenotypes in mice with the Rps9 D95N mutation [48], contribute little to the accumulation of pathogenic Aβ proteins, levels of phosphorylated tau and plaque deposition.

Our results are perhaps surprising, given the previously suspected causative role of age-related proteostasis decline in the accumulation of Aβ in sporadic AD. However, while many chaperones are active anti-aggregants in vitro [54], it has been difficult to parse out their active biological roles during the NDD process. Apparently contradictory animal studies of chaperone overexpressing and knock-out mutants in NDD mice models point to an incomplete understanding of the fundamental proteostatic mechanisms involved in beta amyloid deposition [13, 26, 29, 51, 53].

Several models consistently indicate a decline in proteostasis and an accumulation of misfolded proteins during aging [25, 31, 41, 50]. More recently, an accelerated development of age-related pathologies was demonstrated in Rps9 D95N *ram* mice, which exhibit elevated levels of error-prone translation and an increased propensity for protein aggregation [48]. In addition, together with increased formation of protein aggregates in the hippocampus, the D95N mice showed clinical symptoms and neuronal dysfunction reminiscent of early human AD, namely cognitive impairment, maladaptive emotional responses, epileptic seizures, sleep perturbation, and glucose hypometabolism [6]. Further to increased formation of protein aggregates, transcriptional enrichment of GO terms related to ubiquitin-dependent proteasomal degradation and protein folding in brain of D95N mice points to perturbations of protein homeostasis in these animals. We here addressed the question whether in addition to these findings, the misfolding-prone environment present in the D95N mice affects accumulation and aggregation of pathogenic Aβ proteins. Surprisingly, we found that proteostasis decline, as present in the D95N mice with heightened levels of error-prone translation, does not promote accumulation of Aβ nor Aβ plaque formation.

A limitation of the Rps9 D95N mouse model is that the molecular mechanisms and signalling pathways that drive the premature development of age-related pathologies remain to be further characterized in detail, as do a more refined analysis of the proteostasis defects in these mice and detailed biochemical studies of the misfolded proteins. Building upon and extending our previous findings, the data reported here support the idea that age-related accumulation of non-specific misfolded proteins may contribute to NDD pathogenesis independently of the Aβ cascade. In sporadic AD, this combines with the effects of Aβ, which accumulates for other reasons and presumably on a different time scale. Here, a possible clue behind Aβ accumulation points to polymorphisms in apolipoprotein E, which can influence aggregation and clearance of Aβ and are consistently identified as a risk factor for age-related AD [4, 47, 55]. A caveat to our research may be whether the proteostasis decline present in D95N mice is large enough to mirror the proteostasis decline under physiological conditions of aging. However, as the pressure on proteostasis networks in D95N is significant enough to induce premature aging phenotypes [48], we are confident that the previously indicated impact on proteostasis in D95N mice provides a good reason to suppose that we are studying physiologically relevant levels of proteostasis decline.

In order to minimize the total number of animals used, histopathological and biochemical analyses were performed on the same brains. While we realize the limitations of immunohistochemical analysis on unperfused brains, we thrust our results for various reasons. First, they are mirrored and independently supported by results from quantitative measurements of Aβ peptides and tau phosphorylation using ELISA and Western blot. Second, they faithfully and quantitatively reproduce the expected spectrum from weak to very severe immunohistochemical pathology reported in mice carrying the various humanized pathogenic APP genotypes [37, 45]. Third, our immunohistochemical quantification detects and reproduces the exacerbating effect of female sex known to be present in these lines of APP knock-in mice (personal communication by Takashi Saito), [37]. We take these observations as evidence that our study design and methods of neuropathological characterization would be suitable and sensitive enough to detect any relevant exacerbating effect of increased mistranslation on AD-related pathology, if it were present. In this respect, we consider our data as fully conclusive and sufficient to support our conclusion that perturbations of protein homeostasis as mediated by the translation error-prone Rps9 D95N *ram* mutation do not promote Aβ-related pathology.

How the proteostasis network handles the increasing burden of protein aggregates, both during aging and during the development of NDDs associated with the accumulation of specific pathogenic proteins, is a topic of increasing interest [22]. From our data, we conclude that while heightened levels of error-prone translation result in the accelerated development of several age-related pathologies and increase the propensity for random protein aggregation, they contribute little to the formation of Aβ aggregates. We propose that an age-related decline in proteostasis plays an important role in NDD aetiology, through the accumulation of randomly misfolded proteins, which have more recently been shown to exert detrimental effects on neuronal function similar to, but independent of, Aβ.

### Supplementary Information

Below is the link to the electronic supplementary material.

Supplementary file1 (PDF 2364 KB) Supplementary Figure 1. Humanization of the APP exon DNA sequence surrounding amyloid beta. Synonymous codons in mouse and human APP were identified, often with divergent codon usage frequency. Numbers represent codon usage frequency per 1000 codons. Blue boxes indicate synonymous codons in mice which were altered to match the human sequence. Green boxes indicate the G-R, F-Y and R-H amino acid substitutions to humanize the mouse APP amino acid sequence. Red boxes show the positions of the amyloidogenic mutations (NL, G, F). The positions of APP cleavage by beta and gamma secretases are also labelled. The two cleavage positions for gamma secretase produce amyloid beta 40 or amyloid beta 42. Supplementary Figure 2. Generation of humAPP mutant mice. (a) RT-PCR results from different expression vectors for APP mRNA transfected into NIH-3T3 cells. CTRL: Non-transfected cells, Exp1: wild-type mouse APP control, Exp2: G676R, F681Y, R684H humanization mutations, Exp3: G676R, F681Y, R684H humanization mutations with synonymous codons, Exp4: G676R, F681Y, R684H humanization mutations with synonymous codons and humanized intronic sequences flanking the humanized exons, Exp5: G676R, F681Y, R684H humanization mutations with synonymous codons and NL mutations, Exp6: G676R, F681Y, R684H humanization mutations with synonymous codons, NL mutations and humanized intronic sequences flanking the humanized exons, Exp7: G676R, F681Y, R684H humanization mutations with synonymous codons and NL-F mutations, Exp8: G676R, F681Y, R684H humanization mutations with synonymous codons, NL-F mutations and humanized intronic sequences flanking the humanized exons, Exp9: G676R, F681Y, R684H humanization mutations with synonymous codons and NL-G-F mutations, Exp10: G676R, F681Y, R684H humanization mutations with synonymous codons, NL-G-F mutations and humanized intronic sequences flanking the humanized exons. The strongest band slightly below 800-bp is the correctly spliced APP. (b) Representative example of the screening of ES cell clones. A fragment was amplified spanning a region within the APP locus and the neighboring region from the correct insert region. One copy of the positive control vector in the presence of 30 ng wild-type C57BL/6N genomic DNA (+WT) served as positive control. The positive control vector mimics the DNA conformation at the targeted locus once the homologous recombination is performed, between the short homology arm of the targeting vector and the app locus. PCR with wild-type DNA (WT) or without DNA (H20) served as negative controls. PCR fragments were separated by capillary electrophoresis using AATI ZAGTM Fragment Analyzer. (c) Schematic representation of APP alleles. Hatched rectangles represent exons and red lines indicate human intronic sequences. loxP sites are represented by blue triangles. A neomycin cassette is represented by a grey rectangle. Diagrams are not depicted to scale. Supplementary Figure 3. The effect of APP NL, NL-F and NL-G-F mutations on amyloid beta accumulation and Tau phosphorylation in mouse cortex. (a-d) Soluble and insoluble amyloid beta measured by ELISA in mouse cortical tissue expressing amyloidogenic APP mutations, for (a) soluble amyloid beta 40 (Aβ40), (b) soluble amyloid beta 42 (Aβ42) (c) insoluble amyloid beta 40 (Aβ40), and (d) insoluble amyloid beta 42 (Aβ42). (8 ≤ N ≤ 12) (Males 4 ≤ N ≤ 7; Females 4 ≤ N ≤ 6). Plots show mean and SD. (e) Representative Western blot images and densitometry analysis of Tau and phosphorylated Tau proteins comparing humAPPWT/WT with humAPPNL-F/NL-F and humAPPNL-G-F/NL-G-F mice (N=8) (males N=4; females N=4). GAPDH was used as loading control. Densitometry depicts relative expression of target gene normalized to loading control. For comparison, the APP WT genotype was set as 1. **p<0.01, ***p<0.001 N = number of mice in each comparison. Supplementary Figure 4. Representative tissue sections illustrating complete absence of Aβ plaques in dorsal hippocampus and neocortex of humAPPNL/NL (a-b) and humAPPWT/WT (c-d) mice without (a, c) and with (b, d) the Rps9 D95N mutation. Arrowheads point at non-specific staining of blood vessels by the Aβ antibody due to the use of tissue from unperfused animals. Giemsa counterstain appears in light blue. Scale bar 200μm (a-d). Supplementary Figure 5. Total APP protein expression levels in mutant mouse cortex. APP in mice harboring humAPP, with or without the Rps9 D95N ram mutation, was compared by Western blot and analyzed by densitometry. Comparisons were made for mice with (a) humAPPWT/WT, (b) humAPPNL/NL and (c) humAPPNL-F/NL-F. GAPDH as loading control. Plots show mean and SD. No differences in APP expression were observed. Supplementary Figure 6. General astrogliosis revealed by GFAP immunolabeling (a-d) and activation of microglia revealed by Iba1 immunolabeling (e-h) in the hilar region of dorsal hippocampus (a, c, e, g) and in neocortex (b, d, f, h). Representative sections of strong (a-b, e-f) and weak (c-d, g-h) are shown. Differences in astroglial activation were larger in the neocortex than in the hippocampus. Arrows point at the surface of neocortex, arrowheads at non-specific staining of blood vessels by the GFAP antibody due to the use of tissue from unperfused animals. Giemsa counterstain appears in light blue. Scale bar 50μm (a-h). Genotypes: (a-g) humAPPWT/WT, (h) humAPPNL-F/NL-F, Rps9 D95N (a, b, e, g), Rps9 WT (c, d, f, h). Supplementary Figure 7. Integrated stress response pathway in the cortex, Western blot and densitometry analysis of eIF2α, phosphorylated eIF2α, GADD34, and BACE1. GAPDH was used as loading control. Densitometry depicts relative expression of target gene normalized to loading control. For comparison, the respective control was set as 1. ***p<0.001. N = number of mice in each comparison. Graphs show mean, SEM, and individual data points. (a) Mice harboring humAPPNL-G-F/NL-G-F were compared with age matched humAPPWT/WT mice, all without the Rps9 D95N mutation. (7 ≤ N ≤ 8) (Males N=4; Females 3 ≤ N ≤ 4). (b) Mice harboring humAPPWT/WT with or without the Rps9 D95N ram mutation (10 ≤ N ≤ 12) (Males N=6; Females 4 ≤ N ≤ 6). (c) Mice harboring humAPPNL-F/NL-F with or without the Rps9 D95N ram mutation (N=10) (Males N=5; Females N=5)

## Data Availability

The authors declare that data supporting the results of this study are available in the article and its supplementary materials. Further resource information is available from the corresponding author upon reasonable request.
