# Supplementary figures and images for: Mistranslation-associated perturbations of proteostasis do not promote accumulation of amyloid beta and plaque deposition in aged mouse brain
Source: Cell Mol Life Sci. 2023 Nov 27;80(12):378. doi: 10.1007/s00018-023-05031-z (PMC10682081; doi:10.1007/s00018-023-05031-z)

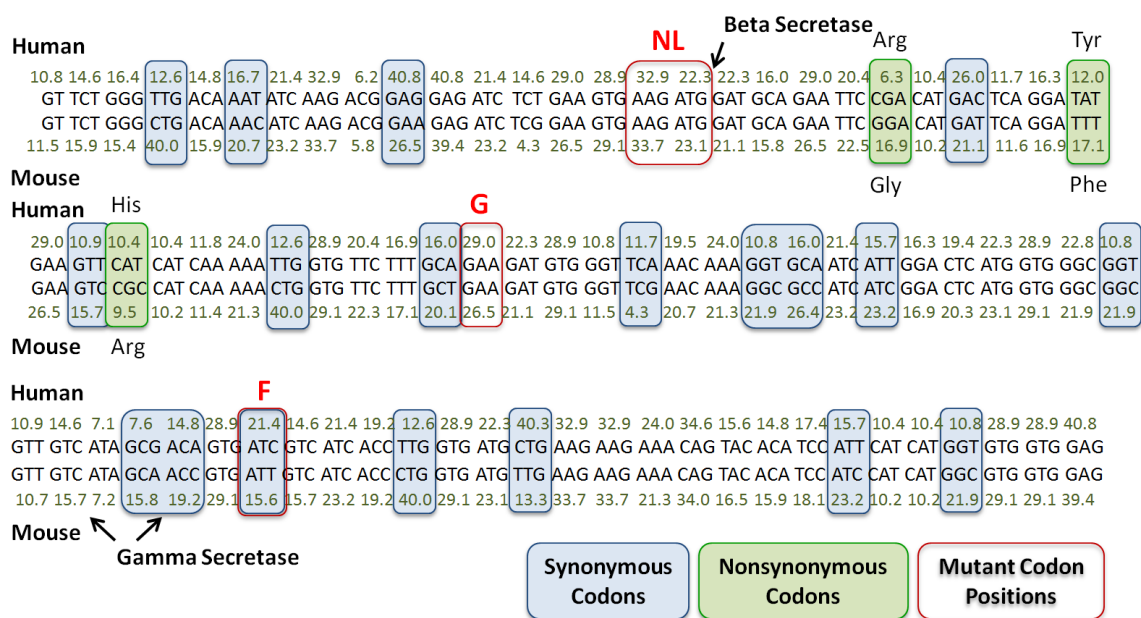

Suppl. Fig. 1

**a**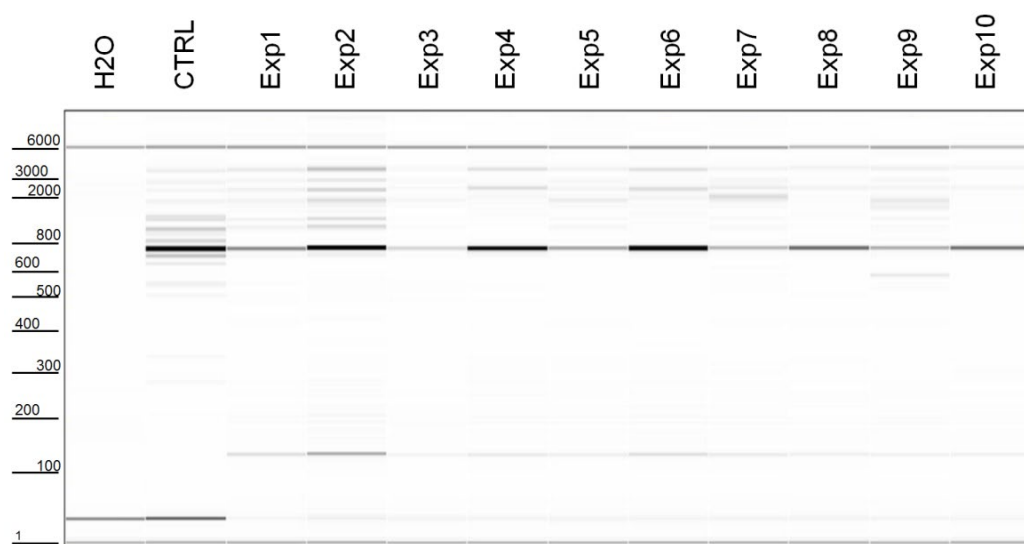**b**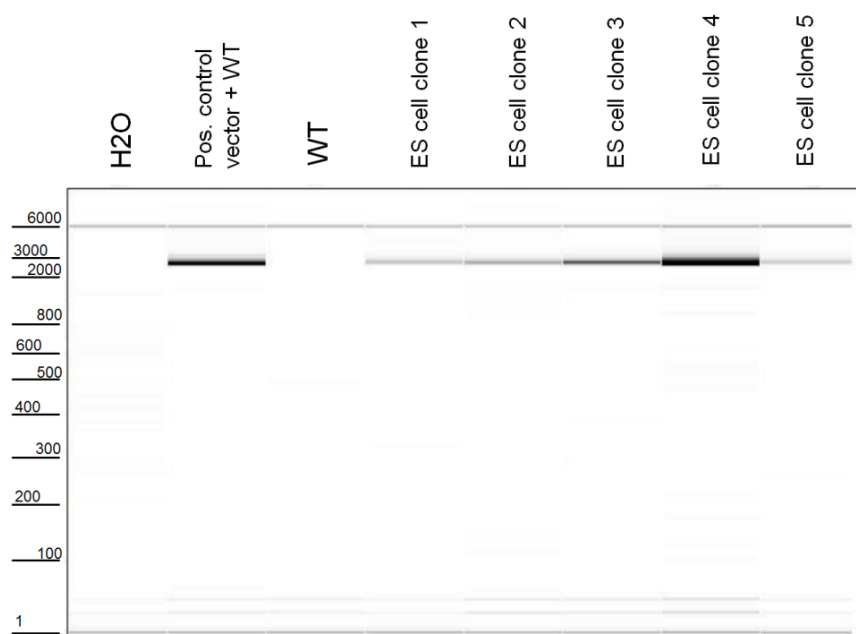**c**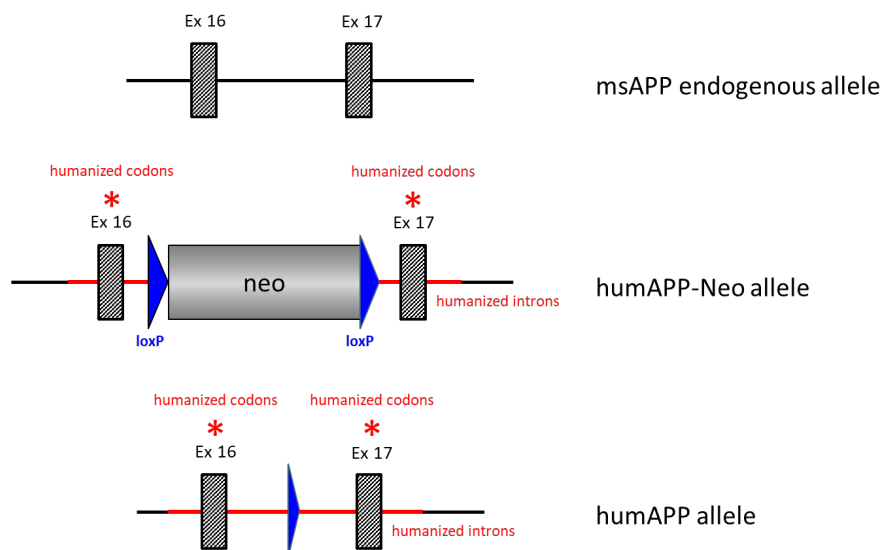

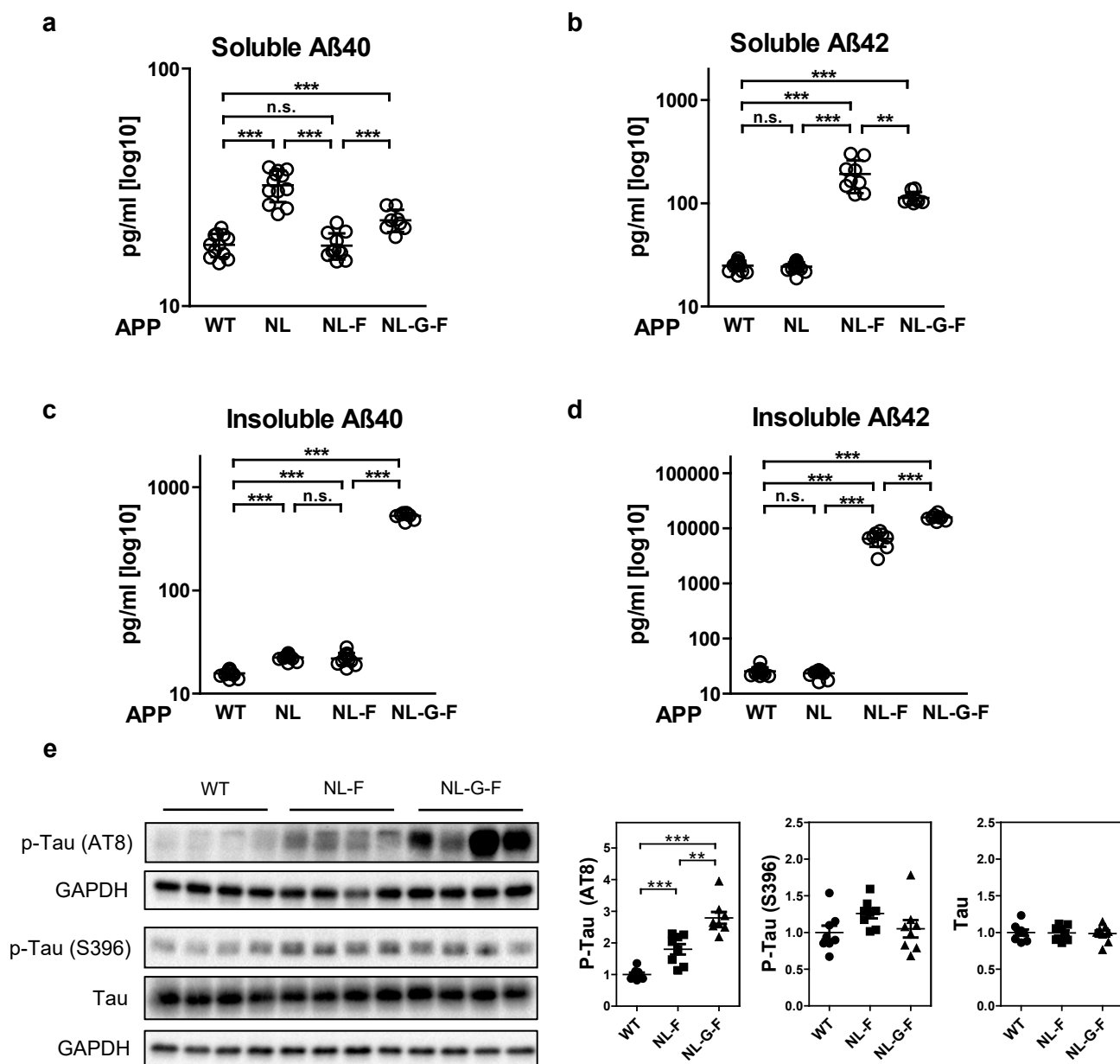

Suppl. Fig. 3

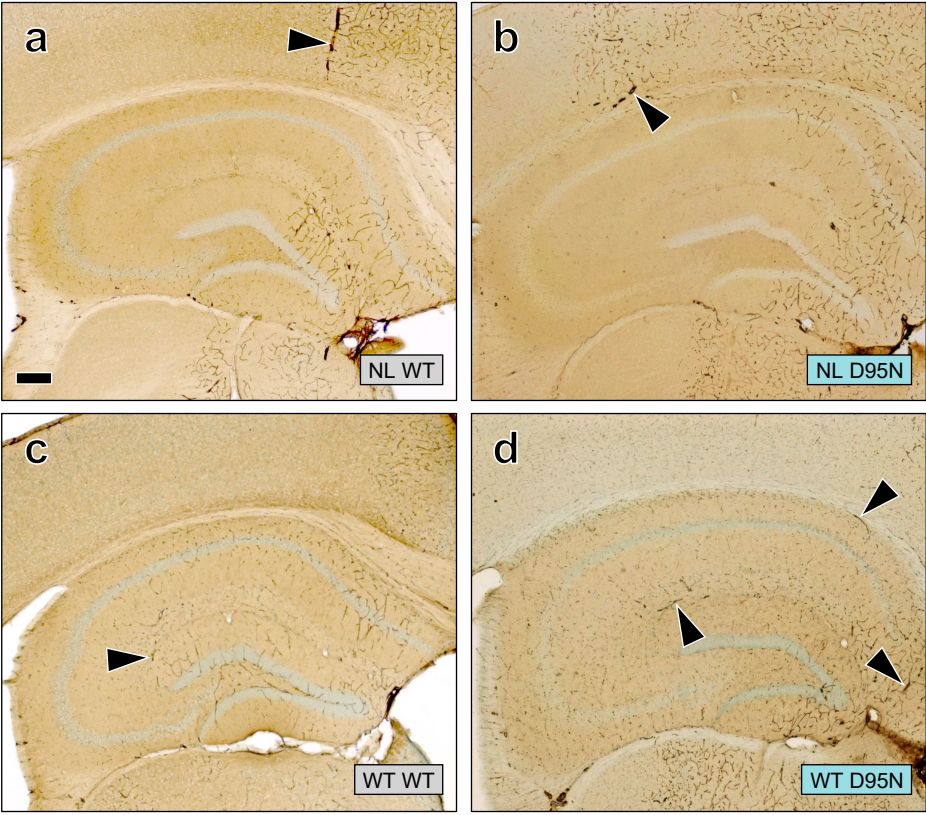

Suppl. Fig. 4

Supplementary Figure 5

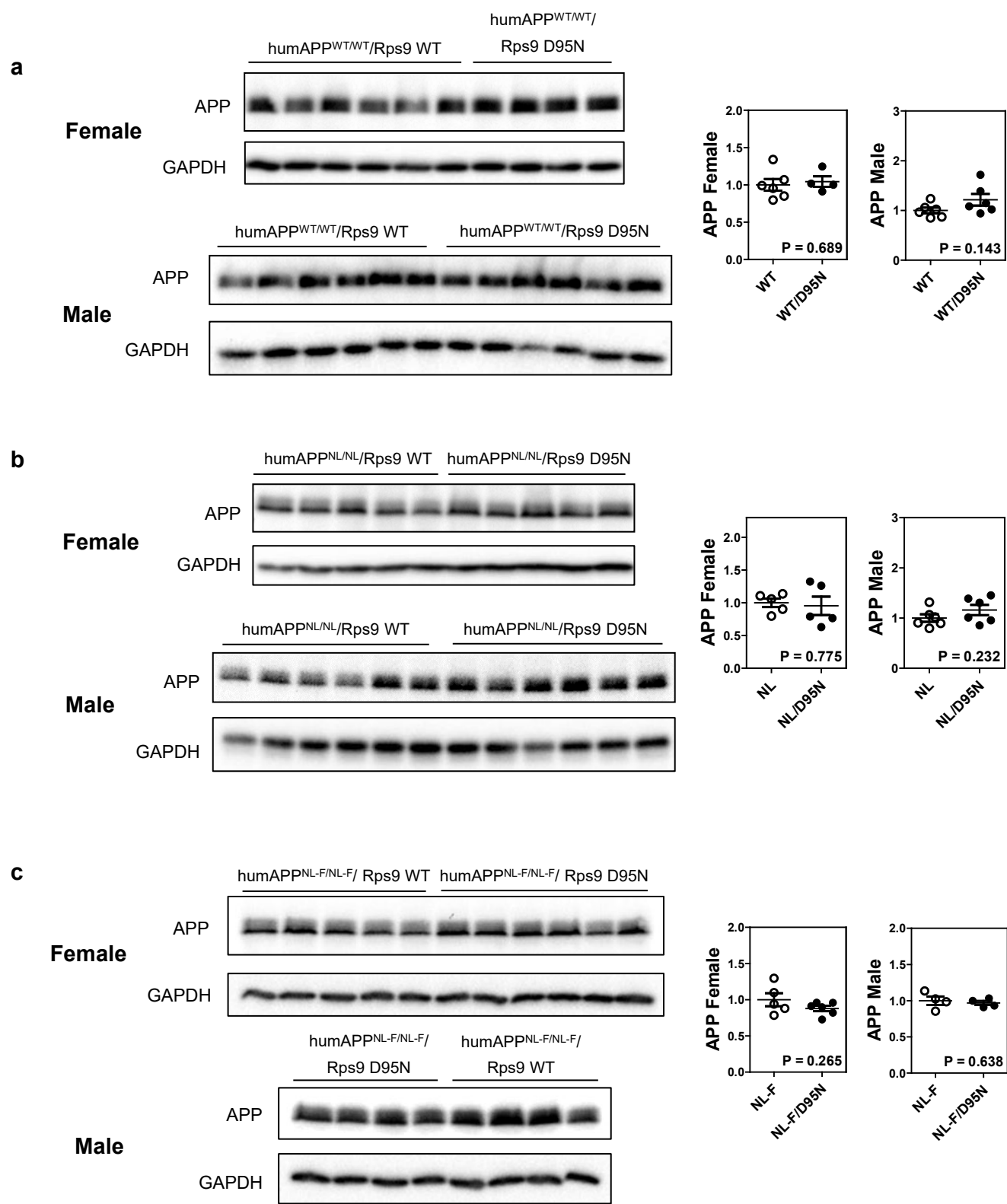

Supl. Fig. 5

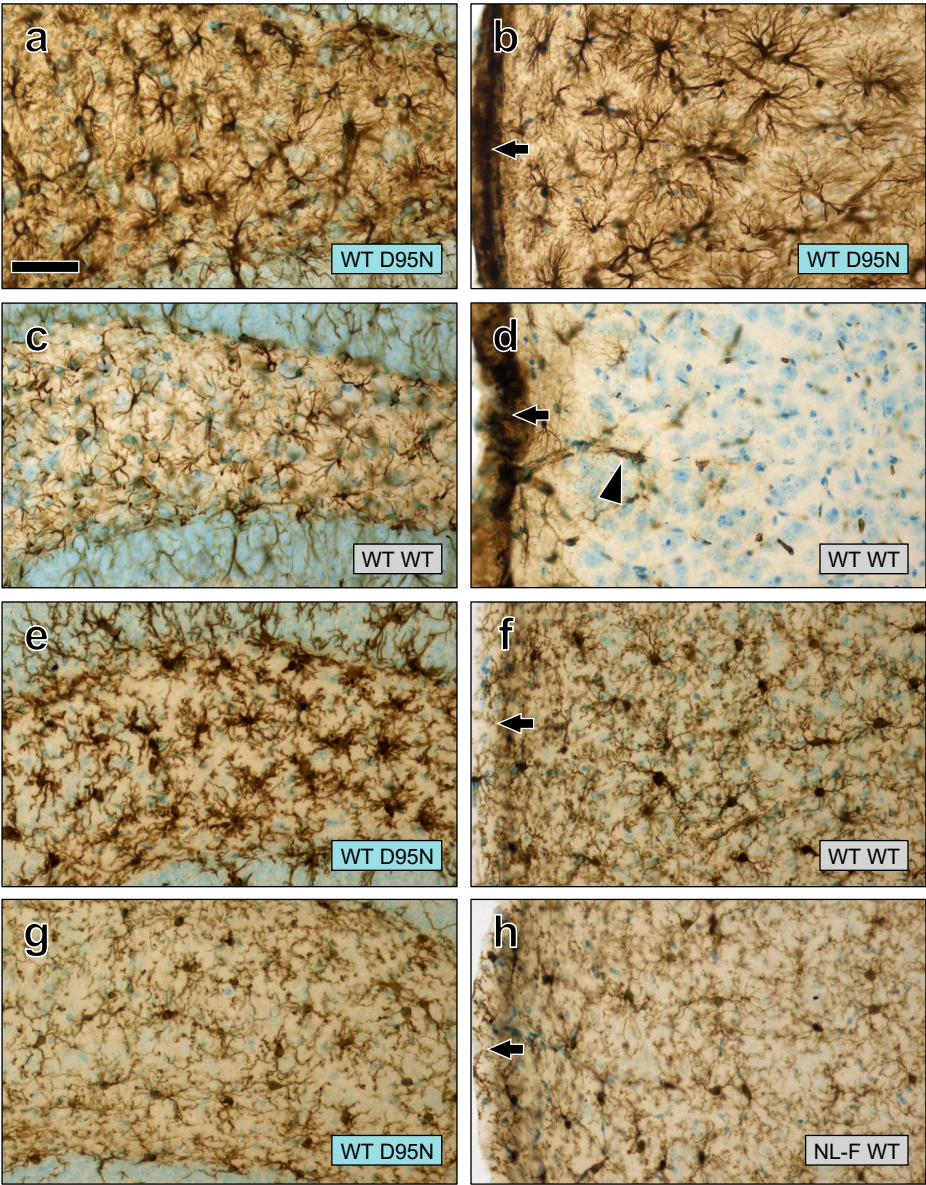

Suppl. Fig. 6

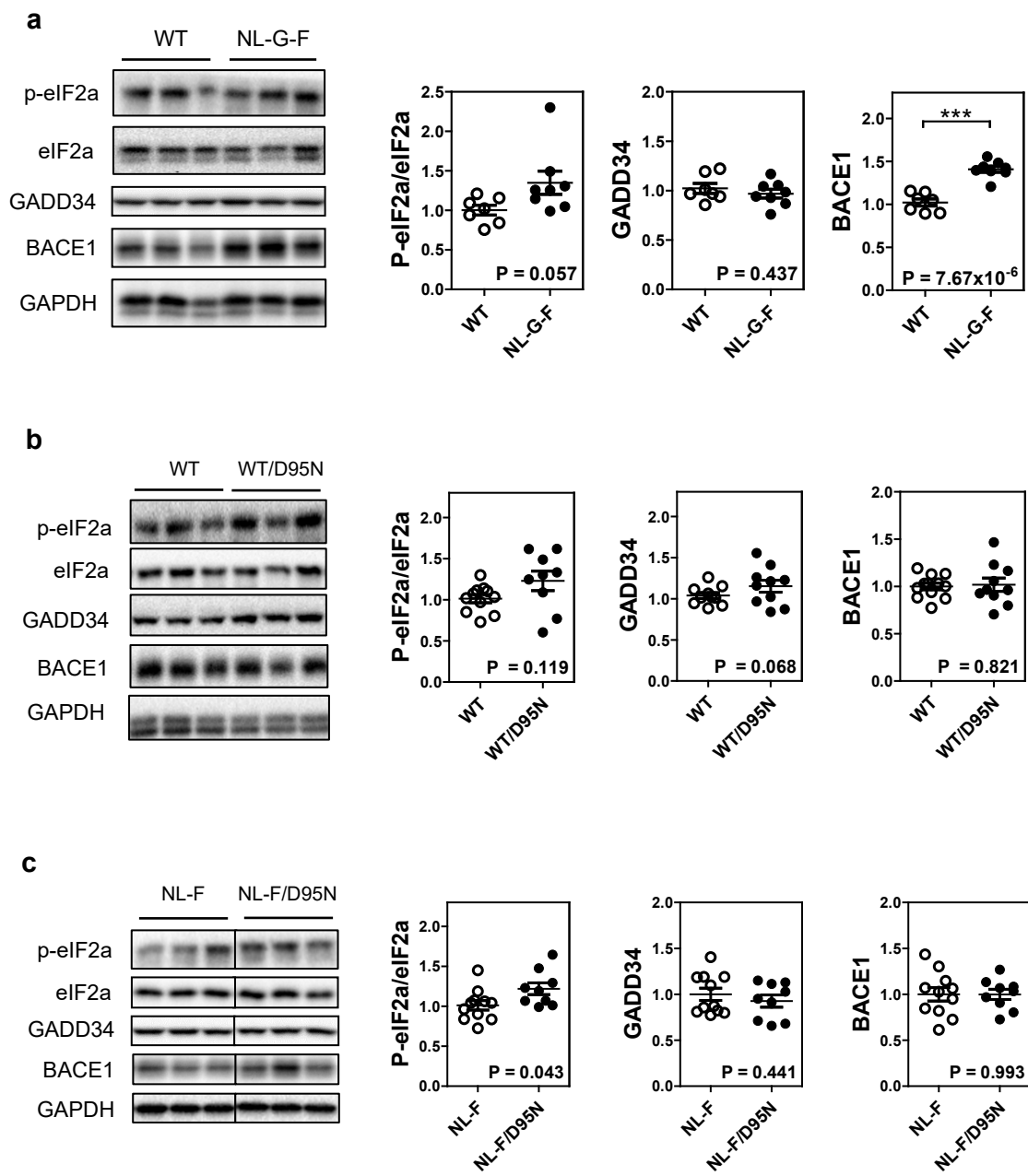

Suppl. Fig. 7

Supplement: Supplementary file 1 — Supplementary file1 (PDF 2364 KB) Supplementary Figure 1. Humanization of the APP exon DNA sequence surrounding amyloid beta. Synonymous codons in mouse and human APP were identified, often with divergent codon usage frequency. Numbers represent codon usage frequency per 1000 codons. Blue boxes indicate synonymous codons in mice which were altered to match the human sequence. Green boxes indicate the G-R, F-Y and R-H amino acid substitutions to humanize the mouse APP amino acid sequence. Red boxes show the positions of the amyloidogenic mutations (NL, G, F). The positions of APP cleavage by beta and gamma secretases are also labelled. The two cleavage positions for gamma secretase produce amyloid beta 40 or amyloid beta 42. Supplementary Figure 2. Generation of humAPP mutant mice. (a) RT-PCR results from different expression vectors for APP mRNA transfected into NIH-3T3 cells. CTRL: Non-transfected cells, Exp1: wild-type mouse APP control, Exp2: G676R, F681Y, R684H humanization mutations, Exp3: G676R, F681Y, R684H humanization mutations with synonymous codons, Exp4: G676R, F681Y, R684H humanization mutations with synonymous codons and humanized intronic sequences flanking the humanized exons, Exp5: G676R, F681Y, R684H humanization mutations with synonymous codons and NL mutations, Exp6: G676R, F681Y, R684H humanization mutations with synonymous codons, NL mutations and humanized intronic sequences flanking the humanized exons, Exp7: G676R, F681Y, R684H humanization mutations with synonymous codons and NL-F mutations, Exp8: G676R, F681Y, R684H humanization mutations with synonymous codons, NL-F mutations and humanized intronic sequences flanking the humanized exons, Exp9: G676R, F681Y, R684H humanization mutations with synonymous codons and NL-G-F mutations, Exp10: G676R, F681Y, R684H humanization mutations with synonymous codons, NL-G-F mutations and humanized intronic sequences flanking the humanized exons. The strongest band slightly below 800-bp is the correctly [file 18_2023_5031_MOESM1_ESM.pdf]
